# Prolonged response following anesthetic-only medial branch blocks: A retrospective analysis of 410 consecutive procedures

**DOI:** 10.1016/j.inpm.2026.100816

**Published:** 2026-08-25

**Authors:** David Sherwood, Jihong Min, Jakob Dovgan, Byron J. Schneider

**Affiliations:** a Department of Orthopaedics, University Health Lakewood Medical Center, Kansas City, MO, USA; b Department of Physical Medicine and Rehabilitation, Vanderbilt University Medical Center, Nashville, TN, USA; c Department of Physical Medicine and Rehabilitation, University of Kansas Medical Center, Kansas City, KS, USA

## Abstract

**Introduction:**

Medial branch blocks (MBBs) are procedures using short-acting local anesthetics to identify candidates for radiofrequency neurotomy (RFN) in suspected facet-mediated pain. Although primarily prognostic tools, some studies report therapeutic responses to MBBs done with or without added corticosteroid, with rates ranging from 11% to over 50%. Even if intended as a diagnostic procedure, prolonged responses to anesthetic-only MBBs, defined as persisting days to months, well beyond the hours-long pharmacokinetic window of the local anesthetic, represent a diagnostic issue to physicians with a poorly defined incidence. This study retrospectively evaluated 410 consecutive MBBs to determine the proportion achieving documented sustained pain relief following anesthetic-only MBBs.

**Methods:**

We retrospectively analyzed 410 consecutive MBBs performed during 2019 at a single academic institution by 4 fellowship-trained physiatrists. All procedures were performed using either 0.25 mL or 0.5 mL of 2% lidocaine or 0.5% bupivacaine. Therapeutic response was defined as meeting all three criteria: ≥80% pain relief calculated from chart records; no progression to repeat block or radiofrequency neurotomy; and explicit clinical documentation of sustained relief as the rationale for halting treatment. Chart review identified patients who did not progress for other reasons including insurance denial, patient preference, loss to follow-up, or alternative treatments. 95% confidence intervals were calculated for therapeutic response rates. Follow-up was retrospective and based on subsequent available medical record review rather than a standardized prospective assessment interval.

**Results:**

410 patients underwent medial branch blocks of the cervical or lumbar spine in the study period. Eight patients (1.95%; 95% CI: 0.85-3.81%) met criteria for prolonged response with documented sustained benefit. Of these, minimum confirmed durations of relief ranged from 21 to 331 days (median 53 days; mean 86.5 days). An additional 11 patients (2.7%) did not progress but lacked documented rationale.

**Conclusions:**

This represents the lowest rate of prolonged pain relief following MBBs reported in published literature. Our findings do not support the premise that routine anesthetic-only MBBs have therapeutic benefit. This study is the first to quantify the incidence of how often a MBB intended for diagnostic purposes halts further treatment due to unexplained pain relief.

## 1 Introduction

Chronic axial spinal pain is a pervasive condition and a leading cause of global disability, with facet joints identified as a potential source of symptoms with prevalence estimates varying by cervical, thoracic, or lumbar region [[1], [2], [3], [4], [5]]. Medial branch blocks (MBBs) are utilized as the clinical standard for prognosticating the likelihood that someone with suspected facet-mediated pain will respond favorably to a radiofrequency neurotomy [2,[6], [7], [8], [9], [10], [11], [12]]. Many consider MBBs exclusively as prognostic tools with effects limited to the pharmacological short duration of the local anesthetic used. Others report that a substantial enough proportion of patients get prolonged relief from MBBs, with or without corticosteroids, with sustained relief ranging from 11% to over 50%, and consider the procedure to also potentially have therapeutic benefit [1,[13], [14], [15], [16], [17], [18], [19], [20]].

Despite published reports suggesting sustained therapeutic benefit from diagnostic MBBs, this finding remains controversial. The International Pain and Spine Intervention Society technical manual and guidelines outlines the role of MBBs as tools for radiofrequency neurotomy candidacy, rather than stand-alone therapeutic interventions [21,22]. Reported therapeutic response rates vary widely across studies with methodological heterogeneity limiting direct comparisons. Furthermore, the limited number of placebo-controlled trials makes it difficult to distinguish true therapeutic effects from natural history, regression to the mean, or placebo responses, which can exceed 30% in chronic pain populations [23,24]. In this context, natural history refers to spontaneous change in symptoms over time independent of intervention, whereas regression to the mean refers to the statistical tendency for unusually severe pain at the time of treatment to be followed by values closer to a patient's average on subsequent assessment.

Moreover, the mechanism by which short-acting anesthetics provide such prolonged analgesia remains a subject of investigation. Typical medications for these procedures may include lidocaine or bupivacaine. Local anesthetics like lidocaine typically have a rapid onset of action, and an expected duration of action between 90 min and 3 h, while longer-acting agents like bupivacaine may last between four and 8 h. Other literature suggests that for medial branch blocks, the typical duration of action may be even less [25]. Because these medications are cleared from the local injection site within hours, pain relief lasting more than 24 h is considered to exceed the traditional pharmacokinetic window of the anesthetic drug alone [[25], [26], [27], [28]].

This retrospective study evaluated patients who underwent diagnostic anesthetic MBBs at a single academic institution to identify the proportion who achieved documented, sustained pain relief sufficient to preclude further intervention, while accounting for alternative explanations for non-progression to subsequent treatment. Reporting this observational data achieves two aims. First, prolonged pain relief following MBBs in other literature is touted as evidence of a potentially therapeutic effect and this study provides cohort data as to if this is an observed phenomenon in this practice. These findings can be compared against published research which reports on outcomes of MBBs with or without corticosteroid as a therapeutic treatment. Second, if MBBs are considered as a truly diagnostic only test, it is one of the first publications to report on the incidence of patients who have pain relief following an anesthetic-only MBB of sufficient duration that it obviates the need for progression to the expected therapeutic intervention via radiofrequency neurotomy of the medial branch.

## 2 Methods

### 2.1 Study design

This retrospective cohort study received Institutional Review Board approval (200772). Medical record numbers for all patients who underwent medial branch blocks between January 1, 2019, and December 31, 2019, at a single department within a large academic institution were obtained using Current Procedural Terminology codes 64490, 64491, 64492, 64493, 64494, and 64495. The study period defines the date range for index medial branch blocks; subsequent second medial branch blocks and RFNs were captured regardless of date, including those performed after December 31, 2019.

### 2.2 Patient selection

Inclusion criteria were: (1) first medial branch block performed during the study period, (2) procedure targeted lumbar medial branches (including L5 dorsal ramus) or cervical medial branches (C3-C7 or third occipital nerve), and (3) no prior medial branch block or radiofrequency neurotomy at the treated level in any previous year.

Exclusion criteria were: (1) index medial branch blocks performed outside the specified dates, (2) thoracic medial branches or sacral lateral branches targeted, and (3) procedures coded identically but targeting different structures (e.g., facet joint injections, costovertebral joint injections).

### 2.3 Procedural technique

Four fellowship-trained physiatrists performed all procedures following Spine Intervention Society guidelines current at the time of the study. For cervical procedures, intermittent fluoroscopy was used with anterior-posterior and lateral views to target the medial branch along the articular pillar, with final needle position in the middle 50% of the articular pillar on the lateral view. For lumbar procedures, intermittent fluoroscopy with oblique, anterior-posterior, and lateral views was used to target the medial branch along the neck of the superior articular process. After needle position was confirmed, contrast was injected to exclude vascular uptake, followed by 2% lidocaine or 0.5% bupivacaine in a volume of 0.25 mL or 0.50 mL per targeted medial branch at physician discretion.

### 2.4 Data collection

Five physicians independently reviewed medical records using a standardized data collection form to extract:

• (a) Pre-procedure numeric rating scale (NRS) pain score
• (b) Post-procedure NRS from the procedure note (immediately post-procedure)
• (c) Percentage pain relief documented in the procedure note
• (d) Post-procedure NRS from the associated nursing note
• (e) Percentage pain relief documented in the nursing note
• (f) Post-procedure NRS from patient pain diary at 30 min or 1 h
• (g) Subsequent progression to second medial branch block (MBB2) or radiofrequency neurotomy (RFN)
• (h) Documentation of clinical rationale for non-progression when applicable

### 2.5 Outcome definition

The primary outcome was therapeutic response, which we defined as meeting all three of the following criteria:

1. Achievement of ≥80% pain relief, determined by either:
  o Patient-reported percentage relief ≥80% documented in (c) or (e), OR
  o Calculated percentage relief ≥80%, using the formula: [(pre-procedure NRS) - (lowest post-procedure NRS from b, d, or f)]/(pre-procedure NRS) × 100
2. No progression from MBB1 to MBB2 or from MBB2 to RFN
3. Explicit clinical documentation of sustained pain relief as the rationale for not pursuing further intervention

The 80% threshold was selected based on regional insurance criteria for RFN candidacy and published diagnostic block protocols [29,30]. Per institutional standard of care and consistent with regional payer coverage criteria for facet joint interventions, progression to a confirmatory second block or RFN was contingent upon documented return of the index pain following the diagnostic block [30].

For patients meeting criteria 1 and 2 but lacking criterion 3, detailed chart review within the study timeline was attempted to identify rationales for non-progression (e.g., insurance denial, patient declined further treatment, loss to follow-up, treatment at outside facility, or other factors).

Follow-up was not standardized to a prespecified interval because of the retrospective design. Instead, sustained benefit was defined pragmatically by review of subsequent available medical record documentation after MBB. A case was classified as a therapeutic response only when the chart documented that pain relief persisted sufficiently to serve as the explicit rationale for not pursuing the next planned facet intervention. Patients were not required to have serial follow-up at predefined intervals. Loss to follow-up was assigned when the available record did not permit determination of subsequent clinical trajectory and no alternative documented explanation for non-progression was identified. Duration of relief was defined as the last confirmed clinical encounter in which the patient continued to report pain relief following the index MBB.

### 2.6 Statistical analysis

Therapeutic response rate was calculated as the proportion of patients meeting all three outcome criteria divided by total qualifying MBBs performed. Binomial 95% confidence intervals were calculated. Descriptive statistics were used to summarize procedural outcomes and reasons for non-progression among patients achieving adequate pain relief.

## 3 Results

From January 1, 2019, to December 31, 2019, clinicians performed 410 qualifying MBBs. Among these, 26 patients (6.3%) achieved ≥80% pain relief and did not progress to MBB2 or RFN. Of these 26 cases, only 8 (1.95%; 95% CI: 0.85-3.81%) had explicit clinical documentation describing sustained pain relief as the rationale for halting further treatment progression. The minimum duration of prolonged response in the chart for these 8 patients ranged from 21 to 331 days, with a median confirmed period of relief of 53 days and mean of 86.5 days.

**Fig. 1:**
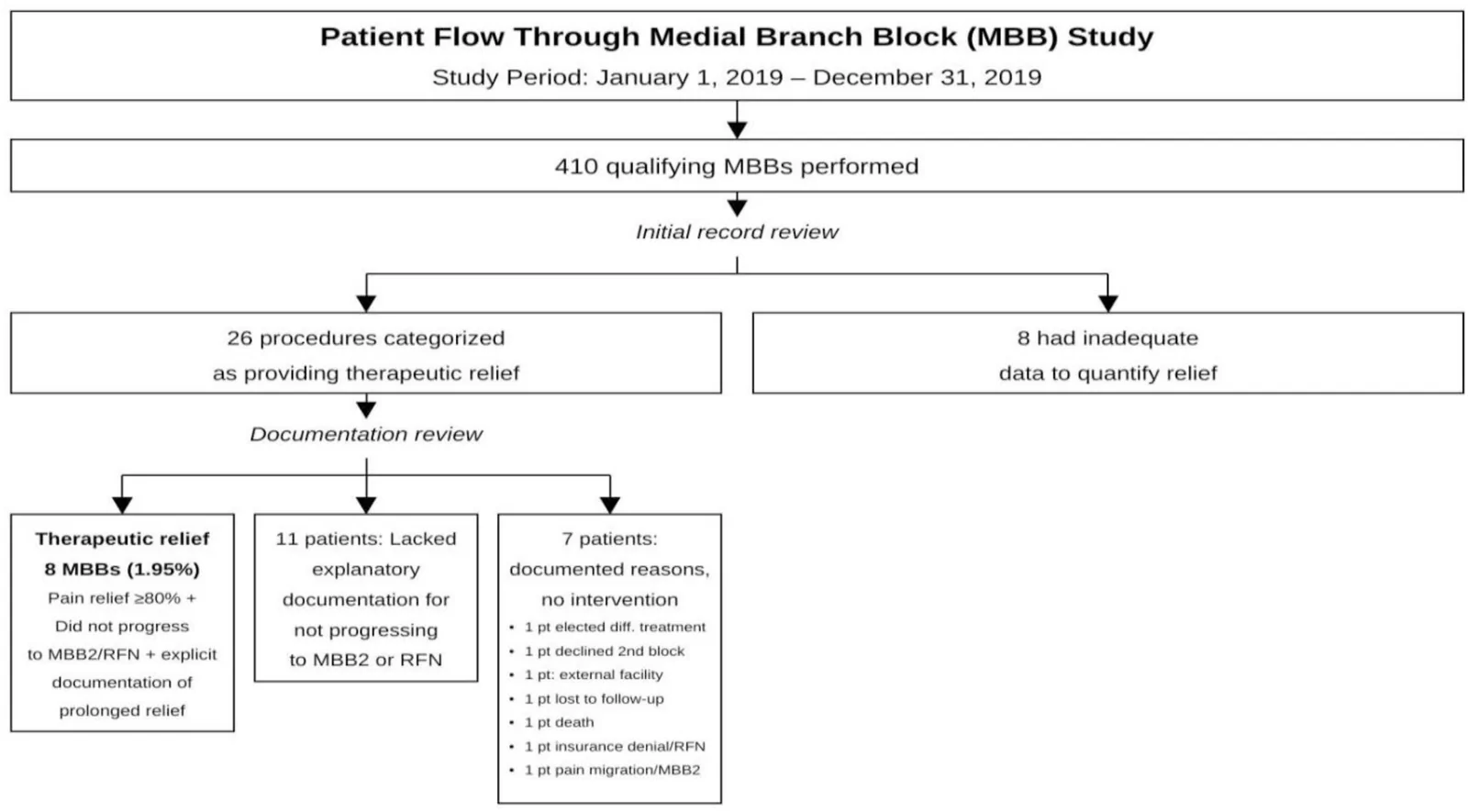
Patient flow through the study. Of 410 qualifying MBBs, 8 met criteria for prolonged therapeutic relief.

Among the remaining 18 patients who achieved adequate relief but did not progress to further intervention, chart review identified the following documented rationales: 1 patient elected an alternative treatment approach, 1 declined repeat block due to procedural discomfort, 1 transferred care to an external facility, 1 was lost to follow-up, 1 died from unrelated causes, 1 experienced insurance denial for RFN, and 1 had pain migration after MBB2 that precluded additional treatment at the originally treated level. Eleven patients (2.7% of total) lacked documented rationale for non-progression despite achieving adequate pain relief. An additional 8 patients of the initial 410 lacked data to quantify their response. See Fig. 1.

## 4 Discussion

This retrospective analysis of 410 consecutive medial branch blocks identified 8 patients (1.95%; 95% CI: 0.85-3.81%) with documented prolonged pain relief following anesthetic-only MBB obviating further intervention. This represents the lowest incidence of prolonged pain relief following MBBs that we were able to identify in the published literature to date. An additional 2.7% (11/410) of patients did not progress to further treatment despite adequate initial pain relief but lacked documentation of the specific rationale for non-progression, making it impossible to attribute non-progression to therapeutic benefit versus other factors such as access barriers, cost, patient preference, or loss to follow-up.

While this study cannot dictate how to manage patients with prolonged relief after a diagnostic MBB, it quantifies a phenomenon relevant to both prevailing views of the procedure. For physicians who use MBBs strictly diagnostically, these cases plausibly represent false-positive responses attributable to placebo effect or other factors, occurring in approximately 1.95% of procedures. Alternatively, viewed as cohort data for a putatively therapeutic intervention, a success rate of 1.95% (95% CI: 0.85–3.81%) does not support the premise that anesthetic-only MBBs performed per current International Pain and Spine Intervention Society (IPSIS) guidelines confer a reasonable expectation of prolonged pain relief [21]. Rather, when stringent outcome criteria are applied and alternative explanations for non-progression are systematically considered, documented sustained benefit is lower than in any other published literature on this topic. Were these considered pilot data, they would not support prioritizing placebo-controlled trials of MBBs as a therapeutic intervention.

Previously published outcomes reporting sustained pain relief following MBBs tout success rates ranging from approximately 11% to over 50% (Table 1) [13,14,16,[18], [19], [20]]. Undoubtedly, skeptics of our findings may point out that the MBBs in this study were performed with anesthetic-only and did not utilize corticosteroids. First, while the effects of corticosteroids may have differing effects, published survey data has reported that only 19.4% of physicians utilize corticosteroids as part of their MBB procedure [31]. Perhaps more importantly though, this simply necessitates closer examination of the other available literature that reports on therapeutic responses to MBBs.

**Table 1:** Published outcomes of prolonged/therapeutic response following medial branch blocks, stratified by injectate.

Study (refs)	Region/design/follow-up	Anesthetic-only MBB outcomes	Anesthetic + corticosteroid MBB outcomes
Manchikanti et al. (14, 31)	Cervical; RCT; 12 and 24 mo	≥50% relief and functional improvement in >83%; 3.5 ± 1.0 procedures at 12 mo; 5.7 procedures at 24 mo	Equivalent NRS/NDI outcomes; 3.4 ± 0.9 procedures at 12 mo; 5.7 procedures at 24 mo
Manchikanti et al. (16, 18)	Thoracic; RCT; 24 mo	15.8 wk relief per procedure; 5.6 procedures; significant NRS/ODI improvement	13.6 wk relief per procedure; 6.2 procedures; equivalent NRS/ODI at all time points
Manchikanti et al. (32)	Cervical; 4-arm RCT; 12 mo	13.4 wk relief per procedure (anesthetic ± sarapin); 3.8 procedures	15.9 wk relief per procedure; 3.5 procedures; equivalent NRS/NDI at all time points
Manchikanti et al. (17)	Additional comparative RCT cohorts by the same group (see note)	16.8 wk relief per procedure; significant NRS/ODI improvement	15.3 wk relief per procedure; equivalent outcomes between groups
Klessinger (13)	Cervical, post-surgical; retrospective; >12 mo	—	53% significant relief without need for additional injections
Liu et al. (19)	Lumbar; retrospective dual-block paradigm; 4 wk after MBB2	Protracted relief in 35% of 146 subjects; 49% of those reporting any initial relief	—
Cohen et al. (35)	Lumbar; RCT vs saline placebo; 1 mo	—	Positive outcome in 11% (vs 6% saline); mean pain reduction 0.7 in both arms
Present study	Cervical and lumbar; retrospective; all available records	1.95% prolonged response (95% CI 0.85–3.81%); median 53 d; mean 86.5 d; range 21–331 d	—

MBB, medial branch block; MBB2, confirmatory second block; RFN, radiofrequency neurotomy; NRS, numeric rating scale; NDI, Neck Disability Index; ODI, Oswestry Disability Index; RCT, randomized controlled trial. Dashes indicate the injectate was not studied in that arm.

Manchikanti et al. reported significant pain relief (≥50%) and functional status improvement observed at 3 months, 6 months, and 12 months in over 83% of patients following cervical MBBs [14]. This study allowed for repeat procedures to be performed in order to reinstate pain relief and was a comparative trial comparing MBBs with anesthetic-only to MBBs with corticosteroids and anesthetic and found no added therapeutic benefit with the addition of corticosteroid. The anesthetic-only group required an average of 3.5 ± 1.0 procedures to achieve these results compared to 3.4 ± 0.9 procedures in the corticosteroid group. 24 month outcomes of this same study were reported, and reported no difference in improvements in NRS pain scores nor NDI measure of disability between the two groups, and again an equal average number of procedures in each group, 5.7, to achieve these outcomes [32]. This study concluded that MBBs with or without corticosteroids were therapeutic.

Manchikanti et al. published a similar randomized comparative study for thoracic MBBs, again finding no added therapeutic benefit of the corticosteroid over anesthetic alone [16,18]. Mean pain scores and ODI were the same in both groups at all time points measured up to 24 months, the average number of procedures required to achieve these results was 5.6 for the anesthetic-only group and 6.2 for the group with added corticosteroid, and the average relief per procedure was 15.8 weeks for the anesthetic-only group vs 13.6 weeks for the corticosteroid group. This study concluded that MBBs with or without corticosteroids were therapeutic.

Another randomized study by Manchikanti et al. compared 4 variations of cervical medial branch blocks involving combinations of anesthetic, sarapin, and steroid with 12 month follow-up [33]. Evaluating the data categorically between the two groups that received corticosteroid and the two groups that did not again found no added benefit of corticosteroid over anesthetic alone. For the group with anesthetic-only or anesthetic with sarapin, the average relief per procedure was 13.4 weeks and the average number of procedures over the study period was 3.8. In the groups with added corticosteroid, the average relief per procedure was 15.9 weeks and the average number of procedures over the study period was 3.5. Improvements in NRS pain scores and NDI scores were also equivalent between groups at all time points. This study concluded that MBBs with or without corticosteroids were therapeutic.

Other comparative studies by the same author group comparing MBBs with anesthetic-only to anesthetic plus corticosteroid consistently reported overall therapeutic benefit of the MBBs but with no difference in outcomes between MBBs done with anesthetic alone vs anesthetic plus corticosteroid. One study again reported significant improvements in NRS and ODI in both groups, with average duration of relief per procedure of 16.8 weeks in the anesthetic-only group vs 15.3 weeks in the group with added corticosteroid. Further enrollment into that same study yielded the similar outcomes, again with no difference between groups [17,18,34].

Collectively, the above studies consistently reported therapeutic benefit from MBBs with decrease in pain and improved function, with average duration of relief per injection typically between 13 and 17 weeks and consistently found no difference between MBBs with anesthetic-only or MBBs with added corticosteroid. Compared to our results, interestingly the documented duration of relief in patients with sustained response is similar to our study which had a mean duration of relief in the 8 patients in which this occurred of just over 12 weeks.

Klessinger reported on MBBs with anesthetic and steroid in patients with persistent neck pain following cervical spine surgery and reported that 53% of patients had significant pain relief following the MBB without the need for additional injections with a follow-up “in excess of 12 months” [13].

Liu et al. reported on lumbar medial branch blocks with anesthetic-only, described as reporting on protracted relief of low back pain after diagnostic MBBs. All patients had the procedure performed twice, three weeks apart. Patients reporting sustained pain relief at the 4-week follow-up after the second MBB were defined as having protracted relief. This occurred in 35% of the 146 subjects overall. 49% of the patients that reported pain relief of any duration after MBB reported sustained relief at the 4 week follow-up [19].

These two studies report markedly different outcomes compared to our study. While the Klessinger study differs in that corticosteroid was used in the MBBs, the high success rate of 53% in this study is very similar to the high success rate of 49% in the study by Liu et al. which utilized anesthetic-only. There are admittedly differences in these studies, including the patient population and follow-up period. As a whole though, these two studies do nothing to contradict the findings reported by Manchikanti et al. that suggest there is no inherent difference when looking at outcomes of MBBs with anesthetic-only compared to MBBs performed with added corticosteroid [17,33,35].

Perhaps the strongest defense against repudiation of our findings on the grounds that results would differ if corticosteroid is added to a MBB comes from a prospective randomized study by Cohen et al. in which two of the groups compared were a group that received lumbar MBB with anesthetic and steroid and a group that received a placebo MBB with saline only [20]. At 1 month follow-up the group that received MBB with anesthetic and steroid had a mean reduction in pain of 0.7, mean improvement in ODI of 3, and positive outcome (defined as 2-point or more decrease in average back pain score coupled with a satisfaction score of 3 out of 5 or higher) in 11% of patients. This was no different compared to the group that received the saline placebo injection, with similarly reported outcomes of mean reduction in pain of 0.7, mean improvement in ODI of 2, and positive outcome in 6% of patients. Herein, a study that finds no difference between MBBs with anesthetic and steroid compared to saline precludes the argument that our study showed such low rates of prolonged significant pain relief because we used anesthetic-only as opposed to anesthetic plus corticosteroid. One might argue that the 11% positive outcome rate observed with anesthetic plus steroid in that study represents a clinically meaningful difference from the 1.95% rate of prolonged response in our cohort. That argument, however, presumes an 11% success rate is an acceptable benchmark for a therapeutic procedure, a premise few clinicians would endorse, particularly when the saline group achieved 6%. Regardless, the Cohen study inherently cannot be used as supporting evidence that adding corticosteroids to MBBs is warranted.

Potential mechanisms for prolonged analgesia beyond pharmacokinetic duration remain speculative. Theories include, but are not limited to, disruption of pain cycles, temporary denervation effects, modulation of central sensitization, or reset of maladaptive pain processing [[36], [37], [38]]. However, these mechanisms remain theoretical. Other possibilities include placebo response, procedural effects to the myofascial tissue, or natural symptom variability. Ultimately, these therapeutic benefits fail to be clinically demonstrable in the vast majority of patients within our cohort.

According to our results, approximately 1 in 50 patients undergoing MBBs with anesthetic-only may achieve sufficient long-term relief to obviate further treatment. This is widely discrepant with the other comparative effectiveness literature that consistently reported significant long-term reductions in pain following MBBs, whether performed with or without corticosteroid. This is one of, if not the only, published studies that corroborates the general premise from the Cohen et al. study suggesting that prolonged therapeutic responses to MBBs do not occur at a rate that exceeds placebo. This study confers clinical context and external validity to those research trial outcomes from Cohen et al. The fact that we were unable to identify any literature that documents outcomes of MBBs with corticosteroids are materially different in terms of long-term pain relief than those of MBBs done with anesthetic alone dispels the notion that our outcomes are invalid because of a lack of corticosteroid utilization.

## 5 Limitations

This study has several limitations which should shape clinician interpretation of the results. First, the retrospective design precluded systematic, protocolized follow-up. Without prospective contact with all non-progressors or retrospective review of documentation of the lack of progression reason, we cannot distinguish therapeutic success from loss to follow-up, access barriers, or patient decision-making unrelated to clinical improvement.

Second, without a control group or sham procedure arm, we cannot determine whether the observed responses represent true treatment effects versus placebo responses, regression to the mean, or natural history. Published literature demonstrates placebo response rates exceeding 30-40% in chronic pain interventions, and our observed 1.95% therapeutic response rate could theoretically be explained entirely by spontaneous improvement or placebo effects [23,24].

Third, we did not control for concurrent interventions including physical therapy, medications, chiropractic care, or behavioral modifications that may have contributed to sustained pain relief independent of the medial branch blocks. Patients experiencing therapeutic benefit may have simultaneously engaged in other effective treatments.

Fourth, our use of the lowest post-procedure NRS value from multiple time points (immediate post-procedure, nursing note, or 30-60 min diary) may have inflated pain relief calculations by capturing transient maximal responses rather than sustained relief. A more conservative approach would have utilized a single, standardized time point beyond the immediate peri-procedural period.

Fifth, our definition of prolonged relief relied on insurance mandated relief as a percentage. Therefore, there may be patients who achieved 50-79% relief and did not achieve progression from MBB1 or MBB2 due to insurance mandated requirements but their relief was sustained and clinically relevant. Our study was not designed to detect these potential responders.

Sixth, documentation varied in completeness across providers and cases. The 11 patients with adequate pain relief but no documented explanation for non-progression represent a source of uncertainty. Without follow-up, we cannot determine their clinical trajectories.

Seventh, our study was conducted at a single academic institution with standardized procedural techniques and a relatively homogeneous practice pattern. Generalizability to community practices, different geographic regions, alternative practice settings, or varied patient populations is uncertain. Additionally, socioeconomic factors, insurance status, and healthcare access barriers were not systematically evaluated.

Eighth, our outcome definition required non-progression. Duration of relief among patients who progressed to MBB2 or RFN was not recorded. Patients who may have experienced prolonged relief followed by recurrence and eventual progression would be classified as non-responders in our study. The reported 1.95% therefore represents a conservative estimate of the incidence of relief exceeding the anesthetic's pharmacokinetic duration, while accurately estimating relief sufficient to halt treatment. Patient-perceived duration of relief following diagnostic MBBs has been characterized prospectively elsewhere [25].

Finally, our follow-up duration was limited to the study period and subsequent available medical records. Patients who experienced delayed recurrence of symptoms after the study period would not have been captured in our analysis.

## 6 Conclusion

This retrospective analysis identified a 1.95% rate of prolonged pain relief following medial branch blocks with anesthetic-only, representing the lowest estimate in published literature. Interestingly, the mean duration of relief in this small subset of patients was consistent with other literature. Literature review does not support the premise that outcomes of MBBs performed with corticosteroids have different “therapeutic” outcomes compared to MBBs with anesthetic alone. For those that continue to view MBBs solely as a diagnostic procedure, this is the first reported estimate of how often patients may experience unexplained prolonged pain relief that negates the need for progressing to radiofrequency neurotomy.
